# Mortality Risk and Survival in the Aftermath of the Medieval Black Death

**DOI:** 10.1371/journal.pone.0096513

**Published:** 2014-05-07

**Authors:** Sharon N. DeWitte

**Affiliations:** Department of Anthropology, University of South Carolina, Columbia, South Carolina, United States of America; University of California, United States of America

## Abstract

The medieval Black Death (*c*. 1347-1351) was one of the most devastating epidemics in human history. It killed tens of millions of Europeans, and recent analyses have shown that the disease targeted elderly adults and individuals who had been previously exposed to physiological stressors. Following the epidemic, there were improvements in standards of living, particularly in dietary quality for all socioeconomic strata. This study investigates whether the combination of the selective mortality of the Black Death and post-epidemic improvements in standards of living had detectable effects on survival and mortality in London. Samples are drawn from several pre- and post-Black Death London cemeteries. The pre-Black Death sample comes from the Guildhall Yard (n = 75) and St. Nicholas Shambles (n = 246) cemeteries, which date to the 11^th^–12^th^ centuries, and from two phases within the St. Mary Spital cemetery, which date to between 1120-1300 (n = 143). The St. Mary Graces cemetery (n = 133) was in use from 1350–1538 and thus represents post-epidemic demographic conditions. By applying Kaplan-Meier analysis and the Gompertz hazard model to transition analysis age estimates, and controlling for changes in birth rates, this study examines differences in survivorship and mortality risk between the pre- and post-Black Death populations of London. The results indicate that there are significant differences in survival and mortality risk, but not birth rates, between the two time periods, which suggest improvements in health following the Black Death, despite repeated outbreaks of plague in the centuries after the Black Death.

## Introduction

The Black Death was one of the most devastating epidemics in human history. It was the first outbreak of medieval plague in Europe, and it killed tens of millions of people, an estimated 30–50 percent of the European population, between 1347–1351 [1]–[3]. This massive, extremely rapid depopulation event initiated or enhanced social, demographic, and economic changes throughout Europe, and thus has attracted the interest of a variety of researchers for decades [2], [4], [5]. Previous bioarchaeological research, using individuals buried in the East Smithfield Black Death cemetery from London, examined the selectivity of Black Death mortality, i.e. whether the medieval epidemic targeted particular individuals or whether, as is often assumed given its very high mortality levels, it killed indiscriminately. The results of this research indicated that people varied in their risks of dying during the Black Death [6]–[8]. In particular, older adults appear to have been more likely to die during the epidemic than their younger peers. Analysis of skeletal markers of physiological stress (short adult stature, enamel hypoplasia, tibial periosteal lesions, cribra orbitalia and porotic hyperostosis), which have been shown under conditions of normal, non-epidemic medieval morality to be associated with elevated risks of mortality [8], [9], revealed that people of all ages (not just the elderly) who were already in poor health (i.e. those who had been exposed to physiological stressors and had developed skeletal stress markers as a result) before the Black Death subsequently faced higher risks of death during the epidemic than their healthier peers. Thus, despite its incredibly high levels of mortality, the Black Death was, like most normal causes of death, a selective killer.

Given that the mortality associated with the Black Death was extraordinarily high and selective, the medieval epidemic might have powerfully shaped patterns of health and demography in the surviving population, producing a post-Black Death population that differed in many significant ways, at least over the short term, from the population that existed just before the epidemic. By targeting frail people of all ages, and killing them by the hundreds of thousands within an extremely short period of time, the Black Death might have represented a strong force of natural selection and removed the weakest individuals on a very broad scale within Europe. In particular, given that reproductive-aged individuals with relatively high frailty (i.e. an individual's risk of death relative to other members of the population [10]) were more likely to die during the Black Death than their age-peers with lower frailty, the epidemic might have affected genetic variation with respect to disease susceptibility or immune competence and thus, acted to reduce average levels of frailty in the surviving population. This might explain why, according to historical documents, medieval plague mortality declined steeply between the initial outbreak in 1347–1351 and the second outbreak in 1361 and why mortality levels remained lower in subsequent plague outbreaks throughout the medieval and early modern periods [11]–[13]. Perhaps people who survived the Black Death and their descendants were generally less frail and less likely to die from a variety of causes (including plague) compared to the pre-epidemic population because of heightened immune responses or reduced disease susceptibility, i.e. traits that were selectively favored during the epidemic. If this was the case, it is possible that the Black Death had positive (though perhaps short-lived) effects on population-level patterns of health and survival.

Observed decreases in mortality levels during medieval plague epidemics after the Black Death might reflect molecular changes in the pathogen responsible for the epidemic, and subsequent plague outbreaks, that rendered it less virulent rather than reflecting changes in health and susceptibility within the human host population. Recent molecular analyses of bone and tooth samples of people who died during the Black Death have yielded DNA from the causative pathogen of the medieval epidemic, *Yersinia pestis* (which continues to affect human populations today by causing bubonic plague) [14]–[19]. By comparing the genome of ancient *Y. pestis* to that of modern strains of the bacterium, such molecular investigations have the potential to reveal the genetic determinants of epidemiological changes in the disease over time. However, a recently published draft genome of 14^th^-century *Y. pestis* did not reveal any unique derived positions in the ancient strain compared to a modern reference strain in regions of the genome associated with virulence [16]. Further analyses of medieval *Y. pestis* may yet reveal functionally significant differences between historic and modern plague. However, given that pathogen virulence is the result of host-pathogen interaction and not simply a characteristic of the pathogen itself, the current lack of evidence of such genetic differences might mean that factors other than, or in addition to, evolution of the pathogen itself were at play in the changing epidemiology of plague [20], [21].

In addition to its potential as a selective agent operating upon intrinsic biological factors, the Black Death might also have shaped population patterns by severely altering exogenous factors that affected health and demography. Historical documents from the post-Black Death period indicated that standards of living improved after the epidemic, at least in some areas of Europe such as England. These changes in standards of living resulted in large part from the massive depopulation caused by the Black Death, which reversed the pre-epidemic conditions of an excess population relative to resources [22]. After the Black Death, there was a severe shortage of laborers, effectively ending the medieval system of serfdom, and consequently wages improved dramatically while prices for food, goods, and housing fell [23]. These changes represented a major redistribution of wealth. Real wages rose to levels that were not exceeded until the 19^th^ century, which allowed for improvements in housing and diet for people of all social status levels [1], [24]–[28]. In England, for example, grain prices dropped steeply after 1375 and generally remained low for almost a century and a half thereafter [29]. Though it took several years for real wages to rise in England in the aftermath of the Black Death (in fact, they may have actually dropped in the period immediately after the epidemic), by the late 14^th^ century real wages had risen sharply to their medieval peak [30]. By the late 15^th^ century, real wages were at least three times higher than they had been at the beginning of the 14^th^ century [29]. The shortage of labor presented new freedoms to workers and placed new pressures on employers. Given that the number of workers was not only smaller than had existed before the Black Death, but that they had new opportunities for mobility and alternative employment if they found existing conditions unsatisfactory, employers increased not only wages but also payments in kind, such as extra food and clothing, to attract workers [23].

Improvements in diet after the Black Death, and particularly decreases in social inequities in diet that presumably benefitted the majority of the lower status population of England, might have acted to reduce average levels of frailty in the population, perhaps more than any other factor associated with improvements in standards of living. Changes in diet can lead to changes in health because nutritional status strongly influences immune competence [31]. Following the Black Death, the amount of money spent per capita on food increased, and people ate higher quantities of relatively high-quality wheat bread, meat, and fish, much of which was consumed fresh rather than salted as had been common prior to the epidemic [29]. Such changes probably improved the nutritional quality of the diet [29], and given that the diet of lower classes became more similar to that of high status individuals, a greater proportion of the post-Black Death English population was consuming a nutritious diet than had been true before the epidemic.

This study examines whether the selective mortality of the Black Death, combined with consequent rising standards of living after the epidemic, resulted in a healthier post-epidemic population in London compared to the pre-Black Death population. Given that death is the ultimate outcome of poor health, and that life-expectancy and mortality levels are commonly used indicators of the general health of living populations, this study examines the temporal changes, from the pre- to the post-Black Death periods (1000–1300 *vs*. 1350–1538), in survival and the hazard of mortality (as proxies for health) in London. If people were less frail (healthier) on average after the Black Death than before epidemic, a higher proportion of the post-Black Death population should have survived to older ages compared to the pre-Black Death population. This study also examines temporal changes in overall risks of mortality; if the post-Black Death population was healthier, risks of mortality overall should have declined from the pre- to post-Black Death periods. Previous studies using historical data have examined the demographic consequences of the Black Death; but the results have been mixed, with some finding tentative evidence of improvements in survival and mortality and others finding that survival declined in the centuries following the epidemic [12], [32], [33]. However, as described in the Discussion, these studies suffer from limitations that render any conclusions about population-wide patterns premature; these limitations include pre-Black Death samples that potentially do not fully represent typical pre-Black Death demographic patterns and restriction of analysis primarily to wealthy males.

The bioarchaeological data used in this study allow for assessment of individuals not typically included in medieval documentary data (e.g. the poor and women and children). Though previous modeling work has shown that demographic perturbations, such as the Black Death, can have effects on age-at-death distributions that last for several decades [34], [35], substantial effects are relatively short-lived (i.e. up to 50 years). Given the relatively long time period considered in this study, such potential effects of temporary perturbations in demographic patterns (e.g. as a result of severe famine or other crisis mortality events) are likely not strong enough to affect the conclusions I have drawn in this paper.

## Materials and Methods

### 2.1 Skeletal Samples

#### Ethics statement

All skeletal samples for this study come from medieval London cemeteries and are curated at the Museum of London Centre for Human Bioarchaeology. Site codes and context numbers for each individual used in this study are provided in the supporting information, Table S1. There is no identifying information associated with any of the individuals (i.e. these cemeteries do not have burial records or coffin plates that identify the interred by name), and thus this research does not constitute any risk to living descendants.

Restriction to a single geographic location means that differences between the pre- and post-Black Death samples can be attributed to the effects of the Black Death and changes in standards of living following the epidemic (note, however, that London was a tremendous draw for migrants in this period; the possible effects of migration on the results of this study are addressed in the Discussion).

#### Pre-Black Death Samples: St. Mary Spital, Guildhall Yard, and St. Nicholas Shambles

The pre-Black Death sample comes from three cemeteries, St. Mary Spital, Guildhall Yard, and St. Nicholas Shambles. Guildhall Yard and St. Nicholas Shambles date to the 11–12^th^ centuries, based on stratigraphic and documentary data and artifacts [36]. The main cemetery associated with the hospital and priory of St. Mary Spital has been divided into four periods using Bayesian radiocarbon dating: Period 14 (c. 1120–1200), Period 15 (c. 1200–1250), Period 16 (c. 1250–1400) and Period 17 (c. 1400–1539), and there are both single and multiple burials in each period [37]. A combined random sample of 143 individuals was selected from among the single inhumations (burial type A) from Periods 14 and 15, both of which are believed to predate the use of the cemetery for infirmary burials [37]. This study uses a combined sample of 464 individuals from these three cemeteries; this sample represents all of the individuals in the St. Nicholas Shambles and Guildhall Yard cemeteries that were preserved well enough to provide data on age using the methods described below, as well as the random sample from St. Mary Spital (who were similarly well preserved). The pre-Black Death sample contains a mixture of lower status individuals, high status individuals, and members of religious communities and thus provides an appropriate comparison sample for the post-Black Death sample from St. Mary Graces [37], [38].

#### Post-Black Death Sample: St. Mary Graces

The post-Black Death sample comes from the cemetery associated with the Cistercian Abbey of St. Mary Graces, which was established in London in 1350, soon after the Black Death ended, and it was in use until the Reformation in 1538 [39], [40]. Burials in St. Mary Graces include both high and low status individuals and monks [41]. This study uses a sample of 133 individuals from St. Mary Graces who were preserved well enough to provide data on age using the methods described below.

### 2.2 Age Estimation

To avoid the problems associated with traditional methods of age estimate, such as age mimicry and poor estimates for older adults [42], [43], ages for this study were estimated using the method of transition analysis described by Boldsen et al. [44]. Transition analysis avoids the biases associated with traditional methods of age estimation and provides point estimates of age even for older adults rather than the broad terminal adult age category (e.g. 50+) typical of traditional methods. This latter feature is particularly useful in the current context, as point estimates for older adults allows for the estimation of important trends that may have occurred at the latest adult ages.

In transition analysis, data from a known-age reference collection are used to obtain the conditional probability, *Pr(c_j_|a)*, that a skeleton will exhibit a particular age indicator stage or suite of age indicator stages given the individual's known age. This conditional probability is combined, using Bayes' theorem, with a prior distribution of ages at death to determine the posterior probability that a skeleton in the cemetery sample died at a certain age given that it displays particular age indicator stages. In transition analysis, the prior distribution of ages at death can either be an informative prior based on documentary data or a uniform prior. By combining the conditional probability, *Pr(c_j_|a)*, from a known-age reference sample, with a uniform or informed prior distribution of ages at death, transition analysis avoids imposing the age distribution of the reference sample on the target sample, and is thus preferable to traditional methods of age estimation [44]. For this study, transition analysis was applied to skeletal age indicators on the pubic symphysis and the iliac auricular surface and to cranial suture closure as described by Boldsen et al. [44], and the ADBOU (Anthropological Database, Odense University) Age Estimation software was used to determine individual ages-at-death and the standard errors associated with those point estimates. The ADBOU program uses a conditional probability estimated from the Smithsonian Institution's Terry Collection and an informative prior distribution of ages at death based on data from 17th-century Danish rural parish records.

Ages for subadult individuals (i.e. those individuals for whom all epiphyses had not yet fused) were estimated based on epiphyseal fusion, and dental development and eruption [45]–[50].

### 2.3 Kaplan Meier Survival Analysis

The effect of time period (pre-Black Death = 0, post-Black Death = 1) on survival was assessed using Kaplan-Meier survival analysis with a log-rank test and using pooled data on age from both time periods. Analysis was performed using SPSS version 21.

### 2.4 Hazard Model

The differences in risks of mortality between the pre- and post-Black Death periods were assessed by pooling the adult data from all cemeteries to estimate the Gompertz hazard of mortality and modeling time period (pre-Black Death = 0, post-Black Death = 1) as a covariate affecting the baseline Gompertz hazard of adult mortality. Juvenile mortality is not included in the hazard analysis given the relatively small sample sizes of individuals below the age of 10. The typical human pattern of juvenile mortality (which can be modeled using the negative Gompertz component of the Siler model) starts very high at birth and then declines very rapidly within the first two years of life, and it is difficult to obtain reliable estimates of this mortality pattern without large samples of infants and young children [51]-[53]. The Gompertz hazard is a parsimonious two-parameter model of the typical human pattern of an increasing senescent risk of mortality [51], [54]: 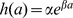. The time period covariate effect was modeled using the proportional hazard specification 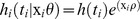
where *h*(*t_i_*) is the baseline Gompertz hazard, *t_i_* is the age of the i^th^ skeleton in years, x*_i_* is the time period covariate, and ρ is the parameter representing the effect of the covariate on the baseline hazard. The advantage of using a hazard model is that it requires the estimation of just a few parameters and thus makes efficient use of the available skeletal data. The model can be applied to relatively small samples, as it smoothes random variation in mortality data without imposing any particular age pattern on the data [54].

Parameters were estimated using maximum likelihood analysis with the program *mle* [55]. A positive or negative estimate for the parameter representing the effect of the time period covariate on the hazard would suggest that those who died in the period following the Black Death faced increased or decreased risk of death, respectively, compared to those who died before the epidemic. The fit of the full model with the time period covariate compared to a reduced model in which the value of the parameter representing the covariate was set equal to 0 was assessed using a likelihood ratio test (LRT). The LRT tests the null hypothesis that time period was not associated with elevated nor decreased risks of death. The LRT was computed as follows: LRT = −2[ln(*L_reduced_*) – ln(*L_full_*)], where LRT approximates a χ^2^ distribution with *df* = 1. Previous research has shown that maximum likelihood estimates are increasingly biased with decreasing sample sizes [56], [57], though maximum likelihood estimation can perform satisfactorily with samples of less than 100 for models with relatively few parameters, such as the Gompertz model used in this study [58]. However, given that the sample sizes used in this study, particularly the post-Black Death sample, are not extremely large, the parameter estimates shown here are useful in so far as they reveal a general trend, but should not be viewed as completely unbiased estimates of the true underlying values.

### 2.5 Fertility Proxy

Given the relatively long period of time across which trends in survival and mortality are examined, this study controls for potential changes in fertility. This is necessary because changes in fertility can alter age-at-death distributions even if age-specific mortality does not change. In fact, cemetery age-at-death distributions are more sensitive to fertility than to mortality, i.e. a change in fertility of a particular magnitude will more dramatically affect age-at-death distributions than will a change in mortality of the same magnitude [59]–[61]. For example, if fertility increases in a population, more children will be born each year than the year before, thus increasing the number of children who die each year, even if age-specific mortality rates remain constant over time. Assuming those children are buried in the same cemetery as the rest of the population, the ever-increasing numbers of young children dying and entering the cemetery will comprise a growing proportion of the associated cemetery over time. Cemeteries are used only for limited periods, such that each birth cohort does not progress completely through the life span before use of the cemetery ceases and thus each birth cohort does not contribute equally to each age interval present in the cemetery. Ultimately, under these circumstances, the resulting cemetery assemblage from this growing population will contain an excess of young individuals relative to older individuals. This phenomenon makes it difficult to infer mortality patterns directly from age-at-death data from cemetery samples [62].

To control for fertility, this study uses the number of the individuals above the age of 30 divided by the number of individuals above the age of 5, i.e. D30+/D5+. Buikstra et al. [63] found that there is a strong (negative) relationship between D30+/D5+ and birth rate, and comparison of the 95% comparison intervals across samples reveals whether birth rates differ significantly among them.

## Results

**Figure 1:**
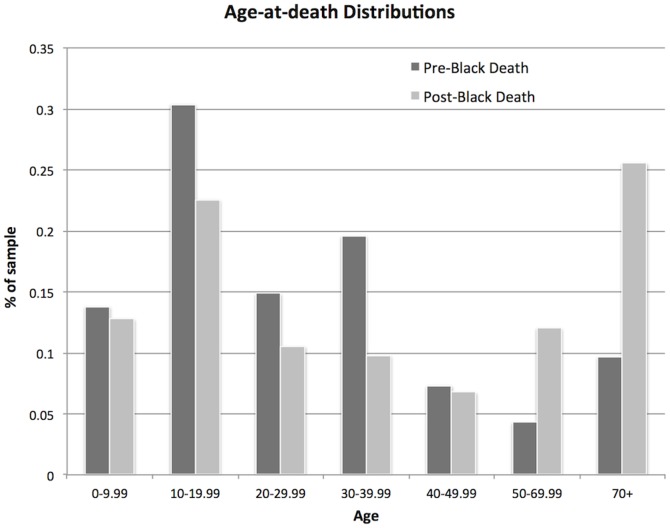
Age-at-death distributions for the pre- and post-Black Death samples.

**Figure 2:**
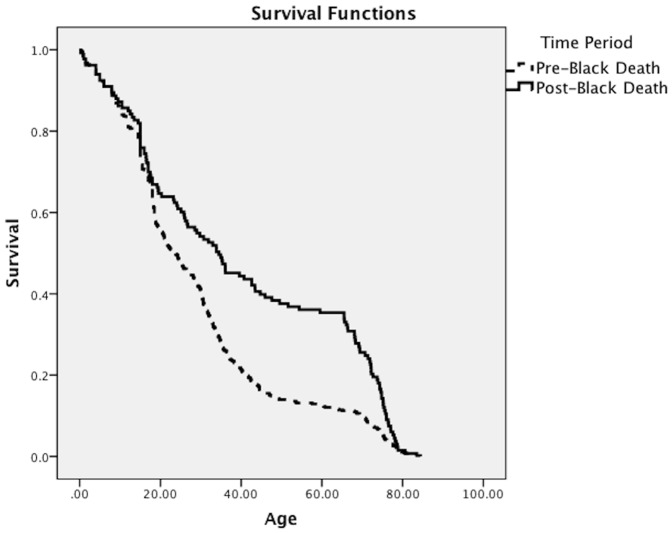
Kaplan-Meier survivorship functions for the pre- and post-Black Death samples.

The pre- and post-Black Death age-at-death distributions, for all ages, are shown in Figure 1; the two distributions are significantly different (Kolmogorov-Smirnov *p*<0.001). In particular, there is a higher proportion of adults above the age of 50 in the post-Black Death sample. The Kaplan-Meier survival curves are shown in Figure 2; the survival functions reveal enhanced survival among those within the post-Black Death sample compared to the pre-Black Death sample (Mantel-Cox *p*<0.001).

The results of the hazard analysis are shown in Table 1. The estimated value of the parameter representing the effect of time period of burial is negative, and the results of the likelihood ratio test indicate that inclusion of the covariate improves the fit of the model. These results indicate reduced risks of mortality across all ages after the Black Death compared to the pre-Black Death population.

**Table 1:** Maximum likelihood estimates (with standard errors) of the Gompertz parameters and the effect of the time period covariate on mortality (with likelihood ratio test).

Parameter	estimate (s.e.)	−2LLR
**α**	0.0096 (0.001)	
**β**	0.034 (0.002)	
**Time period covariate**	−0.57 (0.1)	26.8 (*p*<0.001)

The fertility proxies, i.e. the D_30+_/D_5+_ values and their 95% comparison intervals, are shown in Table 2. The the D_30+_/D_5+_ value for the post-Black Death sample is higher than that of the pre-Black Death sample, which might indicate that birth rates declined following the epidemic. This is consistent with the modeling results found by Paine [34], which indicated a drop in crude birth rates for over 50 years following a catastrophic mortality event. However, as seen in Table 2, the comparison intervals for the two periods overlap, which indicates a lack of a significant difference in birth rates between the two periods [63].

**Table 2:** D_30+_/D_5+_ values and their 95% confidence intervals for the pre- and post-Black Death samples.

	D_30+_/D_5+_	95% CI
**Pre-Black Death**	0.44	0.36–0.51
**Post-Black Death**	0.54	0.45–0.62

## Discussion

The age-at-death distributions from the pre- and post-Black Death samples suggest that survival improved following the Black Death, as the post-Black Death sample has a higher proportion of older adults. This is confirmed by the Kaplan-Meier survivorship analysis, which reveals a higher survivorship function for the post-Black Death sample. The results of hazard analysis indicate that overall, risks of mortality were lower in the post-Black Death population than they were before the epidemic. The D_30+_/D_5+_ values indicate that the observed changes in age-at-death distributions and mortality and survival functions are not an artifact of changes in birth rates between the pre- and post-Black Death periods. Together, these results indicate enhanced survival and improvements in mortality after the Black Death, and by inference, improved health at least at some ages in the post-Black Death population.

The results of this study are consistent with those from some previous studies that focused primarily on documentary evidence. Russell ([12]: p 465) compared post-Black Death, late 14^th^-century age patterns derived from British and Spanish documentary data (tax records and inquisitions post mortem) to the “normal distribution of medieval society by age and sex” derived from skeletal data from 77 medieval burial grounds located primarily in central eastern Europe. His findings suggested that the ratio of individuals above the age of 60 relative to those between ages 20–60 increased after the Black Death in some areas, which would indicate that survival improved following the epidemic. However, there are several limitations to his approach. The dating of the cemeteries included in his study is not clear, but according to Russell, the pooled cemetery sample is more representative of the early medieval period, though it does include the later medieval period. Thus, it appears that he does not have an exclusively pre-Black Death baseline from the skeletal data for comparison with the post-Black Death mortality patterns. The skeletal data were pooled from cemeteries located within a broad geographic region, and thus potentially do not accurately represent normal pre-Black Death mortality patterns for any one particular locale. Further, the geographic areas represented by the skeletal data are mostly eastern and central Europe, and it is not clear whether England and Spain (i.e. the areas for which he had documentary data) are represented at all. This lack of total (or even partial) correspondence in the geographic origin of the skeletal and documentary datasets raises the possibility that the differences observed by Russell reflect population differences rather than the effects of the Black Death. Lastly, both datasets are subject to different but equally problematic sources of bias. The documentary data is biased towards adult males; the skeletal ages were estimated using traditional, biased methods that tend to underestimate older adult ages and overestimate younger adult ages [42]. Because of these limitations, it is possible that the tentative evidence observed by Russell does not truly reflect population-wide patterns resulting from the effects of the Black Death.

To date, the few studies of the demographic effects of the Black Death that have focused exclusively on historical documentary evidence have yielded conflicting results. Nightingale [32] examined records of deaths of creditors (primarily relatively wealthy men) in England from 1300–1530, and found an apparent decrease in “background mortality” following the Black Death. However, these data exclude many members of the population and they begin very soon before the Black Death during a period of severe famine, including the Great Famine 1315–1322 and the resulting Great Bovine Pestilence [64]. Thus, her data might not reflect true baseline, normal pre-Black Death mortality (i.e. they are not an ideal baseline for comparison with the longer post-Black Death period dataset). In contrast to the observed decreases in mortality among wealthy creditors following the Black Death, other studies, of monastic inhabitants, have found that mortality increased for monks in Christ Church Canterbury, Westminster Abbey, and Durham in the 15^th^-century [32], [33]. The patterns observed by Nightingale for wealthy male creditors might more accurately reflect those of the general population than do those of monastic inhabitants, but the fact remains that documentary evidence excludes much of the medieval population, thereby making it difficult to generalize the results of these studies.

This study resolves many of the issues associated with previous studies based on documentary evidence. The bioarchaeological data used in this study allow for the assessment of men, women, and children of various socioeconomic status levels, most of whom are typically missing from many historical documents, and thus the results here are more representative of population-wide demographic patterns. The samples used here are also derived from a period before the Black Death that better represents normal pre-epidemic mortality patterns, for comparison with the post-Black Death data, than the early 14^th^-century data used by Nightingale [32]. The large skeletal samples are from one circumscribed area, thereby reducing the possibility that the observed results reflect population differences rather than the effects of the epidemic. The application of unbiased skeletal age estimation methods means that more nuanced and accurate trends in mortality can be estimated than was possible with the cemetery data in Russell's study [12]. Finally, the use of hazard analysis, which smooths the random variation in data that is inherent in relatively small sample sizes without imposing any particular pattern on the data, allows for evaluation of patterns that are otherwise inaccessible [62]. It should be emphasized that the results of this study should not be viewed as convincing simply because they conform to what one might expect given the evidence available from some historical documents. The bioarchaeological approach should not be considered valuable only in terms of filling in the gaps that exist in historical documents, but rather because it allows us to use data that reflect the experiences of a wider segment of the population to test inferences derived from historical sources [65].

The results of this study are particularly striking given that the Black Death was just the first outbreak of medieval plague, and the period after the epidemic was characterized by repeated crisis mortality resulting in particular from repeated outbreaks of plague. These subsequent outbreaks of medieval plague might have prevented population recovery following the Black Death [5]. Given that catastrophic plague outbreaks were characteristic of the post-Black Death period, but not of the pre-Black Death period considered here, one might reasonably assume that health and survival declined following the Black Death. In London, during the latter half of the 14^th^ century, for example, evidence from wills reveals spikes in mortality associated with plague epidemics in 1361, 1368, 1375, 1382, and 1390 [13]. It should be noted that these sources “exclude married women, children, household servants and apprentices, labourers and paupers” ([13]: p 179), as is true of most medieval sources, so these peaks in mortality might not reflect patterns within the general population if there was any variation by age, sex, or social status. Nonetheless, for at least one segment of the population, the post-Black Death period was characterized by periodic crisis mortality. However, despite repeated plague outbreaks, and other episodes of crisis mortality caused by factors such as famines, the results of this study indicate that the general population enjoyed a period of at least 200 years during which mortality and survival overall improved compared to the pre-Black Death conditions.

The evidence from this study that survival and mortality were affected in positive ways by the Black Death raise the question of what was the proximate cause of these changes. Were the demographic changes a direct result of the selectivity of the epidemic, i.e. the selecting out of frail individuals such that the surviving population was less frail because of inherent biological factors? Or were improvements in diet and other standards of living more important causal factors in these demographic trends? These two possibilities are not necessarily mutually exclusive, as both were ultimately the results of the massive mortality caused by the Black Death. Nonetheless, the disentanglement of intrinsic *versus* extrinsic factors that could have led to changes in survival and mortality is relevant to an understanding, more generally, of the effects of disease on human biology and social, political, and economic conditions. Such a disentanglement, however, requires further study, such as an analysis of stable isotopes and skeletal stress markers that are associated with nutritional deficiencies before and after the Black Death to determine if diet changed in substantial ways that are discernible from the skeleton. Analysis of nitrogen isotope values, for example, might reveal whether people in general consumed substantially more animal protein following the Black Death than was true before the epidemic [66].

The results of this study also raise questions about the possible effects of migration, i.e. whether the pre- and post-Black Death patterns are artifacts of migration into the London after the epidemic rather than, or in addition to, reflecting the effects of the Black Death itself. The improvements in mortality and survivorship observed in the post-Black Death sample might indicate that migration into the city after the epidemic introduced a large number of healthy people. According to Dyer [29] migration likely increased after Black Death as an expression of resistance against restrictions enacted under labor laws in England, such as attempts to prevent increases in wages after 1349. However, London drew substantial numbers of migrants from throughout England and beyond throughout the medieval period, both before and after the Black Death [67], [68]. Famine, in particular, spurred migration before the Black Death, and London attracted large numbers of rural residents in search of work and charity [67]. The existence of migration throughout the medieval period means that both the pre-Black Death and post-Black Death assemblages likely contained a mixture of immigrants and native Londoners, so differences between the two cannot be attributed only to the effects of migration. Furthermore, the possibility of increased immigration to London following the Black Death does not explain the differences in age-at-death distributions observed between the two time periods, the most striking of which is the higher proportion of older adults in the post-Black Death sample. The differences in the age-at-death distributions would reflect the effects of migration alone only if the majority of migrants were older adults; however, it is more likely that most migrants were late teens or young adults [32], [69]. Lastly, the Black Death spared few regions within Europe [70]. Therefore, the selective mortality that operated during the epidemic would have shaped health and demography throughout the continent. Migrants into London in the post-Black Death period would have included survivors of the epidemic and their descendants (and the health and demographic characteristics thereof) to the same or similar extent as did the native population of London. In summary, the existence of migration into London following the Black Death does not necessarily undermine the conclusions made here about positive changes in mortality and, by inference, health following and resulting from the Black Death, thought it is certainly an important issue worthy of further study.

## Conclusions

The results of this study indicate that mortality and survivorship improved in the generations following the Black Death, and that the patterns observed are not simply an artifact of temporal changes in fertility. These results highlight the power that infectious diseases have to shape population-wide patterns of health and demography over both the short- and long-term.

## Supporting Information

Table S1 **Site codes and contexts for all individuals included in the study.** All individuals are curated at the Museum of London Centre for Human Bioarchaeology.
(DOCX)
