# Supplementary material for: Mortality Risk and Survival in the Aftermath of the Medieval Black Death
Source: PLoS One. 2014 May 7;9(5):e96513. doi: 10.1371/journal.pone.0096513 (PMC4013036; doi:10.1371/journal.pone.0096513)
Supplement: Table S1 — Site codes and contexts for all individuals included in the study. All individuals are curated at the Museum of London Centre for Human Bioarchaeology. (DOCX) [file pone.0096513.s001.docx]

**Table S1: Site codes and contexts for all individuals included in the study. All individuals are curated at the Museum of London Centre for Human Bioarchaeology.**

| **Site Name** | **Site Code** | **Context** |
| --- | --- | --- |
| St. Nicholas Shambles | GPO75 | 5062 |
| St. Nicholas Shambles | GPO75 | 5250 |
| St. Nicholas Shambles | GPO75 | 5227 |
| St. Nicholas Shambles | GPO75 | 5314a |
| St. Nicholas Shambles | GPO75 | 5129 |
| St. Nicholas Shambles | GPO75 | 5130 |
| St. Nicholas Shambles | GPO75 | 5212 |
| St. Nicholas Shambles | GPO75 | 5254 |
| St. Nicholas Shambles | GPO75 | 5143 |
| St. Nicholas Shambles | GPO75 | 5095 |
| St. Nicholas Shambles | GPO75 | 5057 |
| St. Nicholas Shambles | GPO75 | 5243a |
| St. Nicholas Shambles | GPO75 | 5210 |
| St. Nicholas Shambles | GPO75 | 5247 |
| St. Nicholas Shambles | GPO75 | 5171a |
| St. Nicholas Shambles | GPO75 | 5125a |
| St. Nicholas Shambles | GPO75 | 5136 |
| St. Nicholas Shambles | GPO75 | 5208 |
| St. Nicholas Shambles | GPO75 | 5140b |
| St. Nicholas Shambles | GPO75 | 5041 |
| St. Nicholas Shambles | GPO75 | 5234 |
| St. Nicholas Shambles | GPO75 | 5014.1 |
| St. Nicholas Shambles | GPO75 | 5029 |
| St. Nicholas Shambles | GPO75 | 5066 |
| St. Nicholas Shambles | GPO75 | 5228 |
| St. Nicholas Shambles | GPO75 | 5214 |
| St. Nicholas Shambles | GPO75 | 5322 |
| St. Nicholas Shambles | GPO75 | 5295 |
| St. Nicholas Shambles | GPO75 | 5170 |
| St. Nicholas Shambles | GPO75 | 5221 |
| St. Nicholas Shambles | GPO75 | 5090 |
| St. Nicholas Shambles | GPO75 | 5138 |
| St. Nicholas Shambles | GPO75 | 5196 |
| St. Nicholas Shambles | GPO75 | 5103 |
| St. Nicholas Shambles | GPO75 | 5119 |
| St. Nicholas Shambles | GPO75 | 5043 |
| St. Nicholas Shambles | GPO75 | 5128 |
| St. Nicholas Shambles | GPO75 | 5058 |
| St. Nicholas Shambles | GPO75 | 5035 |
| St. Nicholas Shambles | GPO75 | 5000 |
| St. Nicholas Shambles | GPO75 | 5259 |
| St. Nicholas Shambles | GPO75 | 5859 |
| St. Nicholas Shambles | GPO75 | 5260 |
| St. Nicholas Shambles | GPO75 | 5193 |
| St. Nicholas Shambles | GPO75 | 5098 |
| St. Nicholas Shambles | GPO75 | 5310 |
| St. Nicholas Shambles | GPO75 | 5318 |
| St. Nicholas Shambles | GPO75 | 5075 |
| St. Nicholas Shambles | GPO75 | 5313 |
| St. Nicholas Shambles | GPO75 | 5204 |
| St. Nicholas Shambles | GPO75 | 5217c |
| St. Nicholas Shambles | GPO75 | 5081 |
| St. Nicholas Shambles | GPO75 | 5152 |
| St. Nicholas Shambles | GPO75 | 5253 |
| St. Nicholas Shambles | GPO75 | 5167 |
| St. Nicholas Shambles | GPO75 | 5111 |
| St. Nicholas Shambles | GPO75 | 5223 |
| St. Nicholas Shambles | GPO75 | 5056 |
| St. Nicholas Shambles | GPO75 | 5080 |
| St. Nicholas Shambles | GPO75 | 5169 |
| St. Nicholas Shambles | GPO75 | 5308 |
| St. Nicholas Shambles | GPO75 | 5137 |
| St. Nicholas Shambles | GPO75 | 5140a |
| St. Nicholas Shambles | GPO75 | 5006 |
| St. Nicholas Shambles | GPO75 | 5161 |
| St. Nicholas Shambles | GPO75 | 5280 |
| St. Nicholas Shambles | GPO75 | 5172 |
| St. Nicholas Shambles | GPO75 | 5244.1 |
| St. Nicholas Shambles | GPO75 | 5092 |
| St. Nicholas Shambles | GPO75 | 5126 |
| St. Nicholas Shambles | GPO75 | 5173 |
| St. Nicholas Shambles | GPO75 | 5028 |
| St. Nicholas Shambles | GPO75 | 5147 |
| St. Nicholas Shambles | GPO75 | 5191 |
| St. Nicholas Shambles | GPO75 | 5019 |
| St. Nicholas Shambles | GPO75 | 5047 |
| St. Nicholas Shambles | GPO75 | 5123 |
| St. Nicholas Shambles | GPO75 | 5217 |
| St. Nicholas Shambles | GPO75 | 5094 |
| St. Nicholas Shambles | GPO75 | 5304 |
| St. Nicholas Shambles | GPO75 | 5109 |
| St. Nicholas Shambles | GPO75 | 5120 |
| St. Nicholas Shambles | GPO75 | 5065 |
| St. Nicholas Shambles | GPO75 | 5068 |
| St. Nicholas Shambles | GPO75 | 5237 |
| St. Nicholas Shambles | GPO75 | 5108 |
| St. Nicholas Shambles | GPO75 | 5083 |
| St. Nicholas Shambles | GPO75 | 5293 |
| St. Nicholas Shambles | GPO75 | 5182 |
| St. Nicholas Shambles | GPO75 | 5222 |
| St. Nicholas Shambles | GPO75 | 5164 |
| St. Nicholas Shambles | GPO75 | 5021 |
| St. Nicholas Shambles | GPO75 | 5217.2 |
| St. Nicholas Shambles | GPO75 | 5225 |
| St. Nicholas Shambles | GPO75 | 5070 |
| St. Nicholas Shambles | GPO75 | 5086 |
| St. Nicholas Shambles | GPO75 | 5067 |
| St. Nicholas Shambles | GPO75 | 5131 |
| St. Nicholas Shambles | GPO75 | 5309 |
| St. Nicholas Shambles | GPO75 | 5134 |
| St. Nicholas Shambles | GPO75 | 5166 |
| St. Nicholas Shambles | GPO75 | 5165 |
| St. Nicholas Shambles | GPO75 | 5203 |
| St. Nicholas Shambles | GPO75 | 5205 |
| St. Nicholas Shambles | GPO75 | 5059 |
| St. Nicholas Shambles | GPO75 | 5122 |
| St. Nicholas Shambles | GPO75 | 5053 |
| St. Nicholas Shambles | GPO75 | 5189 |
| St. Nicholas Shambles | GPO75 | 5125 |
| St. Nicholas Shambles | GPO75 | 5093 |
| St. Nicholas Shambles | GPO75 | 5091 |
| St. Nicholas Shambles | GPO75 | 5118 |
| St. Nicholas Shambles | GPO75 | 5063 |
| St. Nicholas Shambles | GPO75 | 5294 |
| St. Nicholas Shambles | GPO75 | 5003 |
| St. Nicholas Shambles | GPO75 | 5241 |
| St. Nicholas Shambles | GPO75 | 5255 |
| St. Nicholas Shambles | GPO75 | 5022 |
| St. Nicholas Shambles | GPO75 | 5073 |
| St. Nicholas Shambles | GPO75 | 5150 |
| St. Nicholas Shambles | GPO75 | 5239 |
| St. Nicholas Shambles | GPO75 | 5303 |
| St. Nicholas Shambles | GPO75 | 5168 |
| St. Nicholas Shambles | GPO75 | 5240 |
| St. Nicholas Shambles | GPO75 | 5072 |
| St. Nicholas Shambles | GPO75 | 5194 |
| St. Nicholas Shambles | GPO75 | 5206 |
| St. Nicholas Shambles | GPO75 | 5124 |
| St. Nicholas Shambles | GPO75 | 5002 |
| St. Nicholas Shambles | GPO75 | 5183 |
| St. Nicholas Shambles | GPO75 | 5089 |
| St. Nicholas Shambles | GPO75 | 5148 |
| St. Nicholas Shambles | GPO75 | 5112 |
| St. Nicholas Shambles | GPO75 | 5096 |
| St. Nicholas Shambles | GPO75 | 5195 |
| St. Nicholas Shambles | GPO75 | 5316 |
| St. Nicholas Shambles | GPO75 | 5156 |
| St. Nicholas Shambles | GPO75 | 5163 |
| St. Nicholas Shambles | GPO75 | 5213 |
| St. Nicholas Shambles | GPO75 | 5211 |
| St. Nicholas Shambles | GPO75 | 5085 |
| St. Nicholas Shambles | GPO75 | 5207 |
| St. Nicholas Shambles | GPO75 | 5064 |
| St. Nicholas Shambles | GPO75 | 5238 |
| St. Nicholas Shambles | GPO75 | 5101 |
| St. Nicholas Shambles | GPO75 | 5104 |
| St. Nicholas Shambles | GPO75 | 5116 |
| St. Nicholas Shambles | GPO75 | 5244 |
| St. Nicholas Shambles | GPO75 | 5102 |
| St. Nicholas Shambles | GPO75 | 5202 |
| St. Nicholas Shambles | GPO75 | 5144 |
| St. Nicholas Shambles | GPO75 | 5224 |
| St. Nicholas Shambles | GPO75 | 5218 |
| St. Nicholas Shambles | GPO75 | 5012 |
| St. Nicholas Shambles | GPO75 | 5038 |
| St. Nicholas Shambles | GPO75 | 5050 |
| St. Nicholas Shambles | GPO75 | 5192 |
| St. Nicholas Shambles | GPO75 | 5311 |
| St. Nicholas Shambles | GPO75 | 5200 |
| St. Nicholas Shambles | GPO75 | 5159 |
| St. Nicholas Shambles | GPO75 | 5154 |
| St. Nicholas Shambles | GPO75 | 5082 |
| St. Nicholas Shambles | GPO75 | 5178 |
| St. Nicholas Shambles | GPO75 | 5249 |
| St. Nicholas Shambles | GPO75 | 5242 |
| St. Nicholas Shambles | GPO75 | 5257 |
| St. Nicholas Shambles | GPO75 | 5198 |
| St. Nicholas Shambles | GPO75 | 5201 |
| St. Nicholas Shambles | GPO75 | 5039 |
| St. Nicholas Shambles | GPO75 | 5188 |
| St. Nicholas Shambles | GPO75 | 5054 |
| St. Nicholas Shambles | GPO75 | 5216 |
| St. Nicholas Shambles | GPO75 | 5230 |
| St. Nicholas Shambles | GPO75 | 5027 |
| St. Nicholas Shambles | GPO75 | 5133 |
| St. Nicholas Shambles | GPO75 | 5079 |
| St. Nicholas Shambles | GPO75 | 5141 |
| St. Nicholas Shambles | GPO75 | 5209 |
| St. Nicholas Shambles | GPO75 | 5052 |
| St. Nicholas Shambles | GPO75 | 5236 |
| St. Nicholas Shambles | GPO75 | 5215 |
| St. Nicholas Shambles | GPO75 | 5149 |
| St. Nicholas Shambles | GPO75 | 5009 |
| St. Nicholas Shambles | GPO75 | 5199 |
| St. Nicholas Shambles | GPO75 | 5008 |
| St. Nicholas Shambles | GPO75 | 5306 |
| St. Nicholas Shambles | GPO75 | 5001 |
| St. Nicholas Shambles | GPO75 | 5074 |
| St. Nicholas Shambles | GPO75 | 5106 |
| St. Nicholas Shambles | GPO75 | 5155 |
| St. Nicholas Shambles | GPO75 | 5190 |
| St. Nicholas Shambles | GPO75 | 5157 |
| St. Nicholas Shambles | GPO75 | 5220 |
| St. Nicholas Shambles | GPO75 | 5187 |
| St. Nicholas Shambles | GPO75 | 5051 |
| St. Nicholas Shambles | GPO75 | 5158 |
| St. Nicholas Shambles | GPO75 | 5296 |
| Guildhall Yard | GYE92 | 20619 |
| Guildhall Yard | GYE92 | 20117 |
| Guildhall Yard | GYE92 | 13110 |
| Guildhall Yard | GYE92 | 20533 |
| Guildhall Yard | GYE92 | 10770 |
| Guildhall Yard | GYE92 | 10862 |
| Guildhall Yard | GYE92 | 13316 |
| Guildhall Yard | GYE92 | 10419.4 |
| Guildhall Yard | GYE92 | 10419.5 |
| Guildhall Yard | GYE92 | 10689 |
| Guildhall Yard | GYE92 | 10856 |
| Guildhall Yard | GYE92 | 20578 |
| Guildhall Yard | GYE92 | 10419.3 |
| Guildhall Yard | GYE92 | 10756 |
| Guildhall Yard | GYE92 | 13366 |
| Guildhall Yard | GYE92 | 20586 |
| Guildhall Yard | GYE92 | 10699 |
| Guildhall Yard | GYE92 | 10652 |
| Guildhall Yard | GYE92 | 20277 |
| Guildhall Yard | GYE92 | 10419.2 |
| Guildhall Yard | GYE92 | 10696 |
| Guildhall Yard | GYE92 | 10419.6 |
| Guildhall Yard | GYE92 | 10419.7 |
| Guildhall Yard | GYE92 | 13209 |
| Guildhall Yard | GYE92 | 10419.1 |
| Guildhall Yard | GYE92 | 10912 |
| Guildhall Yard | GYE92 | 13042 |
| Guildhall Yard | GYE92 | 10890 |
| Guildhall Yard | GYE92 | 10724 |
| Guildhall Yard | GYE92 | 10690 |
| Guildhall Yard | GYE92 | 10682 |
| Guildhall Yard | GYE92 | 13017 |
| Guildhall Yard | GYE92 | 11772 |
| Guildhall Yard | GYE92 | 20543 |
| Guildhall Yard | GYE92 | 10419 |
| Guildhall Yard | GYE92 | 10647 |
| Guildhall Yard | GYE92 | 10891 |
| Guildhall Yard | GYE92 | 10688 |
| Guildhall Yard | GYE92 | 10705 |
| Guildhall Yard | GYE92 | 10957 |
| Guildhall Yard | GYE92 | 10792 |
| Guildhall Yard | GYE92 | 13367 |
| Guildhall Yard | GYE92 | 10894 |
| Guildhall Yard | GYE92 | 20065 |
| Guildhall Yard | GYE92 | 20538 |
| Guildhall Yard | GYE92 | 10731 |
| Guildhall Yard | GYE92 | 10915 |
| Guildhall Yard | GYE92 | 11692 |
| Guildhall Yard | GYE92 | 10786 |
| Guildhall Yard | GYE92 | 20105 |
| Guildhall Yard | GYE92 | 20588 |
| Guildhall Yard | GYE92 | 20109 |
| Guildhall Yard | GYE92 | 17166 |
| Guildhall Yard | GYE92 | 10723 |
| Guildhall Yard | GYE92 | 10734 |
| Guildhall Yard | GYE92 | 20113 |
| Guildhall Yard | GYE92 | 10739 |
| Guildhall Yard | GYE92 | 10650 |
| Guildhall Yard | GYE92 | 10774 |
| Guildhall Yard | GYE92 | 13287 |
| Guildhall Yard | GYE92 | 10656 |
| Guildhall Yard | GYE92 | 20623 |
| Guildhall Yard | GYE92 | 10425 |
| Guildhall Yard | GYE92 | 10695 |
| Guildhall Yard | GYE92 | 20574 |
| St. Mary Spital | SRP98 | 14258 |
| St. Mary Spital | SRP98 | 12995 |
| St. Mary Spital | SRP98 | 25345 |
| St. Mary Spital | SRP98 | 8032 |
| St. Mary Spital | SRP98 | 13698 |
| St. Mary Spital | SRP98 | 7852 |
| St. Mary Spital | SRP98 | 20531 |
| St. Mary Spital | SRP98 | 1338 |
| St. Mary Spital | SRP98 | 3765 |
| St. Mary Spital | SRP98 | 19067 |
| St. Mary Spital | SRP98 | 25297 |
| St. Mary Spital | SRP98 | 10606 |
| St. Mary Spital | SRP98 | 31022 |
| St. Mary Spital | SRP98 | 32452 |
| St. Mary Spital | SRP98 | 14707 |
| St. Mary Spital | SRP98 | 26570 |
| St. Mary Spital | SRP98 | 1323 |
| St. Mary Spital | SRP98 | 27482 |
| St. Mary Spital | SRP98 | 31249 |
| St. Mary Spital | SRP98 | 3676 |
| St. Mary Spital | SRP98 | 30195 |
| St. Mary Spital | SRP98 | 14865 |
| St. Mary Spital | SRP98 | 3571 |
| St. Mary Spital | SRP98 | 29394 |
| St. Mary Spital | SRP98 | 27501 |
| St. Mary Spital | SRP98 | 31392 |
| St. Mary Spital | SRP98 | 30033 |
| St. Mary Spital | SRP98 | 23090 |
| St. Mary Spital | SRP98 | 27544 |
| St. Mary Spital | SRP98 | 30939 |
| St. Mary Spital | SRP98 | 19633 |
| St. Mary Spital | SRP98 | 13929 |
| St. Mary Spital | SRP98 | 19906 |
| St. Mary Spital | SRP98 | 30453 |
| St. Mary Spital | SRP98 | 30259 |
| St. Mary Spital | SRP98 | 30512 |
| St. Mary Spital | SRP98 | 25306 |
| St. Mary Spital | SRP98 | 19915 |
| St. Mary Spital | SRP98 | 30193 |
| St. Mary Spital | SRP98 | 23214 |
| St. Mary Spital | SRP98 | 30168 |
| St. Mary Spital | SRP98 | 3512 |
| St. Mary Spital | SRP98 | 30039 |
| St. Mary Spital | SRP98 | 15044 |
| St. Mary Spital | SRP98 | 29918 |
| St. Mary Spital | SRP98 | 21588 |
| St. Mary Spital | SRP98 | 1405 |
| St. Mary Spital | SRP98 | 1149 |
| St. Mary Spital | SRP98 | 3345 |
| St. Mary Spital | SRP98 | 14373 |
| St. Mary Spital | SRP98 | 3707 |
| St. Mary Spital | SRP98 | 5954 |
| St. Mary Spital | SRP98 | 3715 |
| St. Mary Spital | SRP98 | 14168 |
| St. Mary Spital | SRP98 | 26214 |
| St. Mary Spital | SRP98 | 26292 |
| St. Mary Spital | SRP98 | 25737 |
| St. Mary Spital | SRP98 | 15339 |
| St. Mary Spital | SRP98 | 1841 |
| St. Mary Spital | SRP98 | 17428 |
| St. Mary Spital | SRP98 | 2993 |
| St. Mary Spital | SRP98 | 26231 |
| St. Mary Spital | SRP98 | 29738 |
| St. Mary Spital | SRP98 | 20139 |
| St. Mary Spital | SRP98 | 22488 |
| St. Mary Spital | SRP98 | 25832 |
| St. Mary Spital | SRP98 | 26068 |
| St. Mary Spital | SRP98 | 23134 |
| St. Mary Spital | SRP98 | 15030 |
| St. Mary Spital | SRP98 | 20082 |
| St. Mary Spital | SRP98 | 30536 |
| St. Mary Spital | SRP98 | 25647 |
| St. Mary Spital | SRP98 | 23211 |
| St. Mary Spital | SRP98 | 30488 |
| St. Mary Spital | SRP98 | 19094 |
| St. Mary Spital | SRP98 | 1334 |
| St. Mary Spital | SRP98 | 27457 |
| St. Mary Spital | SRP98 | 1061 |
| St. Mary Spital | SRP98 | 1571 |
| St. Mary Spital | SRP98 | 9588 |
| St. Mary Spital | SRP98 | 20360 |
| St. Mary Spital | SRP98 | 25012 |
| St. Mary Spital | SRP98 | 25303 |
| St. Mary Spital | SRP98 | 7177 |
| St. Mary Spital | SRP98 | 31395 |
| St. Mary Spital | SRP98 | 27341 |
| St. Mary Spital | SRP98 | 22600 |
| St. Mary Spital | SRP98 | 27748 |
| St. Mary Spital | SRP98 | 3172 |
| St. Mary Spital | SRP98 | 22648 |
| St. Mary Spital | SRP98 | 31245 |
| St. Mary Spital | SRP98 | 15049 |
| St. Mary Spital | SRP98 | 3494 |
| St. Mary Spital | SRP98 | 5568 |
| St. Mary Spital | SRP98 | 30824 |
| St. Mary Spital | SRP98 | 19645 |
| St. Mary Spital | SRP98 | 8873 |
| St. Mary Spital | SRP98 | 4929 |
| St. Mary Spital | SRP98 | 25492 |
| St. Mary Spital | SRP98 | 11371 |
| St. Mary Spital | SRP98 | 1955 |
| St. Mary Spital | SRP98 | 11208 |
| St. Mary Spital | SRP98 | 3235 |
| St. Mary Spital | SRP98 | 9198 |
| St. Mary Spital | SRP98 | 14747 |
| St. Mary Spital | SRP98 | 1046 |
| St. Mary Spital | SRP98 | 19363 |
| St. Mary Spital | SRP98 | 14106 |
| St. Mary Spital | SRP98 | 2899 |
| St. Mary Spital | SRP98 | 25787 |
| St. Mary Spital | SRP98 | 3795 |
| St. Mary Spital | SRP98 | 27375 |
| St. Mary Spital | SRP98 | 27137 |
| St. Mary Spital | SRP98 | 3673 |
| St. Mary Spital | SRP98 | 10771 |
| St. Mary Spital | SRP98 | 27193 |
| St. Mary Spital | SRP98 | 19707 |
| St. Mary Spital | SRP98 | 25728 |
| St. Mary Spital | SRP98 | 29654 |
| St. Mary Spital | SRP98 | 33192 |
| St. Mary Spital | SRP98 | 21523 |
| St. Mary Spital | SRP98 | 17496 |
| St. Mary Spital | SRP98 | 22251 |
| St. Mary Spital | SRP98 | 17710 |
| St. Mary Spital | SRP98 | 5932 |
| St. Mary Spital | SRP98 | 3706 |
| St. Mary Spital | SRP98 | 20490 |
| St. Mary Spital | SRP98 | 2687 |
| St. Mary Spital | SRP98 | 19524 |
| St. Mary Spital | SRP98 | 22380 |
| St. Mary Spital | SRP98 | 29684 |
| St. Mary Spital | SRP98 | 12923 |
| St. Mary Spital | SRP98 | 14383 |
| St. Mary Spital | SRP98 | 5224 |
| St. Mary Spital | SRP98 | 25716 |
| St. Mary Spital | SRP98 | 14868 |
| St. Mary Spital | SRP98 | 33167 |
| St. Mary Spital | SRP98 | 13974 |
| St. Mary Spital | SRP98 | 29698 |
| St. Mary Spital | SRP98 | 13313 |
| St. Mary Spital | SRP98 | 9469 |
| St. Mary Spital | SRP98 | 15047 |
| St. Mary Spital | SRP98 | 14616 |
| St. Mary Spital | SRP98 | 7383 |
| St. Mary Spital | SRP98 | 19638 |
| St. Mary Spital | SRP98 | 30506 |
| St. Mary Spital | SRP98 | 1320 |
| St. Mary Spital | SRP98 | 27689 |
| St. Mary Spital | SRP98 | 25525 |
| St. Mary Spital | SRP98 | 27246 |
| St. Mary Spital | SRP98 | 5771 |
| St. Mary Spital | SRP98 | 14571 |
| St. Mary Spital | SRP98 | 14193 |
| St. Mary Spital | SRP98 | 27266 |
| St. Mary Spital | SRP98 | 27001 |
| St. Mary Spital | SRP98 | 5461 |
| St. Mary Spital | SRP98 | 1777 |
| St. Mary Spital | SRP98 | 19472 |
| St. Mary Spital | SRP98 | 3730 |
| St. Mary Spital | SRP98 | 5275 |
| St. Mary Spital | SRP98 | 1070 |
| St. Mary Spital | SRP98 | 1313 |
| St. Mary Spital | SRP98 | 20533 |
| St. Mary Spital | SRP98 | 15599 |
| St. Mary Spital | SRP98 | 21066 |
| St. Mary Spital | SRP98 | 1847 |
| St. Mary Spital | SRP98 | 4830 |
| St. Mary Spital | SRP98 | 25300 |
| St. Mary Spital | SRP98 | 31352 |
| St. Mary Spital | SRP98 | 7665 |
| St. Mary Spital | SRP98 | 9856 |
| St. Mary Spital | SRP98 | 13343 |
| St. Mary Spital | SRP98 | 32293 |
| St. Mary Spital | SRP98 | 25752 |
| St. Mary Spital | SRP98 | 22071 |
| St. Mary Spital | SRP98 | 3770 |
| St. Mary Spital | SRP98 | 15329 |
| St. Mary Spital | SRP98 | 1073 |
| St. Mary Spital | SRP98 | 10274 |
| St. Mary Spital | SRP98 | 20693 |
| St. Mary Spital | SRP98 | 18196 |
| St. Mary Spital | SRP98 | 1164 |
| St. Mary Spital | SRP98 | 32091 |
| St. Mary Spital | SRP98 | 14037 |
| St. Mary Spital | SRP98 | 19521 |
| St. Mary Spital | SRP98 | 27721 |
| St. Mary Spital | SRP98 | 15814 |
| St. Mary Spital | SRP98 | 20579 |
| St. Mary Spital | SRP98 | 30171 |
| St. Mary Spital | SRP98 | 1316 |
| St. Mary Spital | SRP98 | 31008 |
| St. Mary Spital | SRP98 | 20079 |
| St. Mary Spital | SRP98 | 1913 |
| St. Mary Spital | SRP98 | 23383 |
| St. Mary Spital | SRP98 | 17481 |
| St. Mary Spital | SRP98 | 8420 |
| St. Mary Spital | SRP98 | 13892 |
| St. Mary Spital | SRP98 | 12170 |
| St. Mary Spital | SRP98 | 21582 |
| St. Mary Spital | SRP98 | 30847 |
| St. Mary Spital | SRP98 | 27278 |
| St. Mary Spital | SRP98 | 10230 |
| St. Mary Spital | SRP98 | 29554 |
| St. Mary Graces | MIN86 | 13805 |
| St. Mary Graces | MIN86 | 14429 |
| St. Mary Graces | MIN86 | 13711 |
| St. Mary Graces | MIN86 | 12382 |
| St. Mary Graces | MIN86 | 16081 |
| St. Mary Graces | MIN86 | 9392 |
| St. Mary Graces | MIN86 | 16033 |
| St. Mary Graces | MIN86 | 16119 |
| St. Mary Graces | MIN86 | 9376 |
| St. Mary Graces | MIN86 | 9414 |
| St. Mary Graces | MIN86 | 10082 |
| St. Mary Graces | MIN86 | 13887 |
| St. Mary Graces | MIN86 | 9320 |
| St. Mary Graces | MIN86 | 10491 |
| St. Mary Graces | MIN86 | 13913 |
| St. Mary Graces | MIN86 | 13765 |
| St. Mary Graces | MIN86 | 9369 |
| St. Mary Graces | MIN86 | 9313 |
| St. Mary Graces | MIN86 | 14405 |
| St. Mary Graces | MIN86 | 9351 |
| St. Mary Graces | MIN86 | 13762 |
| St. Mary Graces | MIN86 | 10087 |
| St. Mary Graces | MIN86 | 10142 |
| St. Mary Graces | MIN86 | 12432 |
| St. Mary Graces | MIN86 | 9354 |
| St. Mary Graces | MIN86 | 10060 |
| St. Mary Graces | MIN86 | 10240 |
| St. Mary Graces | MIN86 | 10635 |
| St. Mary Graces | MIN86 | 12410 |
| St. Mary Graces | MIN86 | 12427 |
| St. Mary Graces | MIN86 | 12455 |
| St. Mary Graces | MIN86 | 13959 |
| St. Mary Graces | MIN86 | 16302 |
| St. Mary Graces | MIN86 | 16338 |
| St. Mary Graces | MIN86 | 10111 |
| St. Mary Graces | MIN86 | 10443 |
| St. Mary Graces | MIN86 | 9324 |
| St. Mary Graces | MIN86 | 9310 |
| St. Mary Graces | MIN86 | 12345 |
| St. Mary Graces | MIN86 | 14421 |
| St. Mary Graces | MIN86 | 10170 |
| St. Mary Graces | MIN86 | 10218 |
| St. Mary Graces | MIN86 | 10250 |
| St. Mary Graces | MIN86 | 14424 |
| St. Mary Graces | MIN86 | 12400 |
| St. Mary Graces | MIN86 | 16063 |
| St. Mary Graces | MIN86 | 10190 |
| St. Mary Graces | MIN86 | 10488 |
| St. Mary Graces | MIN86 | 16316 |
| St. Mary Graces | MIN86 | 10058 |
| St. Mary Graces | MIN86 | 12331 |
| St. Mary Graces | MIN86 | 9365 |
| St. Mary Graces | MIN86 | 9362 |
| St. Mary Graces | MIN86 | 13872 |
| St. Mary Graces | MIN86 | 10231 |
| St. Mary Graces | MIN86 | 9373 |
| St. Mary Graces | MIN86 | 9411 |
| St. Mary Graces | MIN86 | 10386 |
| St. Mary Graces | MIN86 | 9339 |
| St. Mary Graces | MIN86 | 16328 |
| St. Mary Graces | MIN86 | 13898 |
| St. Mary Graces | MIN86 | 12297 |
| St. Mary Graces | MIN86 | 9383 |
| St. Mary Graces | MIN86 | 12485 |
| St. Mary Graces | MIN86 | 12414 |
| St. Mary Graces | MIN86 | 13666 |
| St. Mary Graces | MIN86 | 9380 |
| St. Mary Graces | MIN86 | 12424 |
| St. Mary Graces | MIN86 | 13935 |
| St. Mary Graces | MIN86 | 12450 |
| St. Mary Graces | MIN86 | 9334 |
| St. Mary Graces | MIN86 | 10177 |
| St. Mary Graces | MIN86 | 13530 |
| St. Mary Graces | MIN86 | 13848 |
| St. Mary Graces | MIN86 | 16098 |
| St. Mary Graces | MIN86 | 9386 |
| St. Mary Graces | MIN86 | 10241 |
| St. Mary Graces | MIN86 | 10801 |
| St. Mary Graces | MIN86 | 13675 |
| St. Mary Graces | MIN86 | 16104 |
| St. Mary Graces | MIN86 | 10145 |
| St. Mary Graces | MIN86 | 13697 |
| St. Mary Graces | MIN86 | 16073 |
| St. Mary Graces | MIN86 | 12356 |
| St. Mary Graces | MIN86 | 16344 |
| St. Mary Graces | MIN86 | 13670 |
| St. Mary Graces | MIN86 | 9403 |
| St. Mary Graces | MIN86 | 13840 |
| St. Mary Graces | MIN86 | 12349 |
| St. Mary Graces | MIN86 | 10420 |
| St. Mary Graces | MIN86 | 12493 |
| St. Mary Graces | MIN86 | 12110 |
| St. Mary Graces | MIN86 | 13678 |
| St. Mary Graces | MIN86 | 10219 |
| St. Mary Graces | MIN86 | 9425 |
| St. Mary Graces | MIN86 | 16102 |
| St. Mary Graces | MIN86 | 16042 |
| St. Mary Graces | MIN86 | 20000 |
| St. Mary Graces | MIN86 | 10270 |
| St. Mary Graces | MIN86 | 12201 |
| St. Mary Graces | MIN86 | 13825 |
| St. Mary Graces | MIN86 | 13975 |
| St. Mary Graces | MIN86 | 10124 |
| St. Mary Graces | MIN86 | 13849 |
| St. Mary Graces | MIN86 | 14432 |
| St. Mary Graces | MIN86 | 12339 |
| St. Mary Graces | MIN86 | 16304 |
| St. Mary Graces | MIN86 | 13831 |
| St. Mary Graces | MIN86 | 9327 |
| St. Mary Graces | MIN86 | 10164 |
| St. Mary Graces | MIN86 | 13622 |
| St. Mary Graces | MIN86 | 13518 |
| St. Mary Graces | MIN86 | 9399 |
| St. Mary Graces | MIN86 | 16009 |
| St. Mary Graces | MIN86 | 13624 |
| St. Mary Graces | MIN86 | 9417 |
| St. Mary Graces | MIN86 | 12403 |
| St. Mary Graces | MIN86 | 13984 |
| St. Mary Graces | MIN86 | 10493 |
| St. Mary Graces | MIN86 | 10070 |
| St. Mary Graces | MIN86 | 9420 |
| St. Mary Graces | MIN86 | 13747 |
| St. Mary Graces | MIN86 | 12005 |
| St. Mary Graces | MIN86 | 12195 |
| St. Mary Graces | MIN86 | 13633 |
| St. Mary Graces | MIN86 | 10287 |
| St. Mary Graces | MIN86 | 10348 |
| St. Mary Graces | MIN86 | 12473 |
| St. Mary Graces | MIN86 | 12497 |
| St. Mary Graces | MIN86 | 13774 |
| St. Mary Graces | MIN86 | 13724 |
| St. Mary Graces | MIN86 | 10159 |
| St. Mary Graces | MIN86 | 16332 |
